# Prognostic Factors and Survival Outcomes in Squamous Cell Carcinoma of the Thyroid: A Surveillance, Epidemiology, and End Results (SEER) Database Analysis

**DOI:** 10.7759/cureus.63326

**Published:** 2024-06-27

**Authors:** Asif Iqbal, Kue T Lee, Abdul Qahar Khan Yasinzai, Ziyang Li, Agha Wali, Bisma Tareen, Israr Khan, Marjan Khan, Asad Ullah

**Affiliations:** 1 Medicine, Mercy Health, Ardmore, USA; 2 Otolaryngology, Augusta University Medical College of Georgia, Augusta, USA; 3 Oncology, Bolan Medical College, Quetta, PAK; 4 Medicine, Texas Tech University Health Sciences Center, Lubbock, USA; 5 Internal Medicine, Bolan Medical College, Quetta, PAK; 6 Medicine, Insight Hospital and Medical Center, Chicago, USA; 7 Internal Medicine, Marshfield Medical Center, Marshfield, USA; 8 Pathology, Texas Tech University Health Sciences Center, Lubbock, USA

**Keywords:** demographics, survival, seer database, rare cancer, primary squamous cell carcinoma of the thyroid

## Abstract

Introduction

Squamous cell carcinoma of the thyroid (SCCT) is a rare, aggressive thyroid cancer distinguished by the emergence of squamous cells due to chronic inflammation or metaplasia. It poses diagnostic and therapeutic challenges, often identified at an advanced stage with a poor prognosis. The rarity of SCCT underscores the necessity for advanced research on effective treatments and diagnostic strategies. The current data utilized the Surveillance, Epidemiology, and End Results (SEER) database to determine the characteristics and outcomes of patients with primary SCCT.

Methods

De-identified data from patients with primary SCCT from 2000 to 2020 were collected using the SEER database. Demographic data, including age, sex, race, income, and housing, and clinical data including tumor size, tumor stage, nodal status, metastases, treatment modality, survival, and the patient’s status, were extracted. Exclusion criteria were patients with unknown outcomes and missing death certificates. A detailed comparison of the two patient cohorts and univariate and multivariate Cox proportional hazard regression survival analyses were conducted.

Results

Among the 159 primary SCCT patients, the median age was 71 ± 21 years, with 83 females (52.2%) and 76 males (47.8%). The median overall follow-up was 6.0 years (4.41-7.59). The majority were White (108, 67.9%), followed by Hispanic (19, 11.9%). The five-year overall survival (OS) of the study group was 17.6% (95% CI = 14.5-20.7). The five-year disease-specific survival (DSS) was 37.6% (95% CI = 32.7-42.5). There was no significant difference based on surgery, chemotherapy, or radiation (p = 0.134). Age, tumor stage, nodal status, and distant metastases were negative prognostic factors. Sex, race, income, and housing were not predictive of survival.

Conclusion

The current study on SCCT highlights a low five-year OS rate of 17.6% and a DSS rate of 37.6%, with no significant difference in survival based on surgery, chemotherapy, or radiation. The negative prognostic factors included age, tumor stage, nodal status, and distant metastases, whereas sex, race, income, and housing did not significantly predict survival outcomes. These findings underscore the critical need for early detection and the development of more effective treatment strategies to manage SCCT.

## Introduction

Squamous cell carcinoma of the thyroid (SCCT) represents a significant clinical challenge due to its aggressive nature and poor prognosis at advanced stages. In the context of thyroid cancer, SCC is particularly rare, accounting for a small fraction of thyroid malignancies [1]. This rarity contributes to the complexity of understanding its pathogenesis, clinical presentation, and optimal management strategies. Typically arising in older adults, with a median age at diagnosis of 71 years, SCC of the thyroid exhibits no strong gender predilection, affecting males and females almost equally [2,3].

Moreover, the etiology of SCCT is not fully understood but is thought to involve chronic inflammation or metaplastic transformation of pre-existing thyroid cells into squamous cells [2,3]. Epidemiologically, SCCT presents a diverse racial distribution, predominantly affecting White populations, followed by Hispanic groups, reflecting broader demographic patterns in cancer incidence [4]. The survival rates for patients diagnosed with this condition are notably low, with a five-year overall survival (OS) rate of only 17.6% and a disease-specific survival (DSS) rate of 37.6% [5]. This highlights the aggressive nature of the disease and the limited efficacy of conventional treatments like surgery, chemotherapy, and radiation, which are not associated with significant survival advantages.

Additionally, factors influencing prognosis include age at diagnosis, the extent of tumor invasion (tumor stage), lymph node involvement (nodal status), and the presence of distant metastases [5]. Interestingly, socioeconomic factors, such as sex, race, income, and housing, have not been identified as significant predictors of survival, suggesting that the biological behavior of SCC transcends social determinants of health [6]. We aimed to investigate clinical behavior, treatment challenges, and avenues for research to improve patient outcomes in SCCT [6,7]. We examined a large cohort of primary SCCT patients using the Surveillance, Epidemiology, and End Results (SEER) database.

## Materials and methods

Data collection

**Figure 1:**
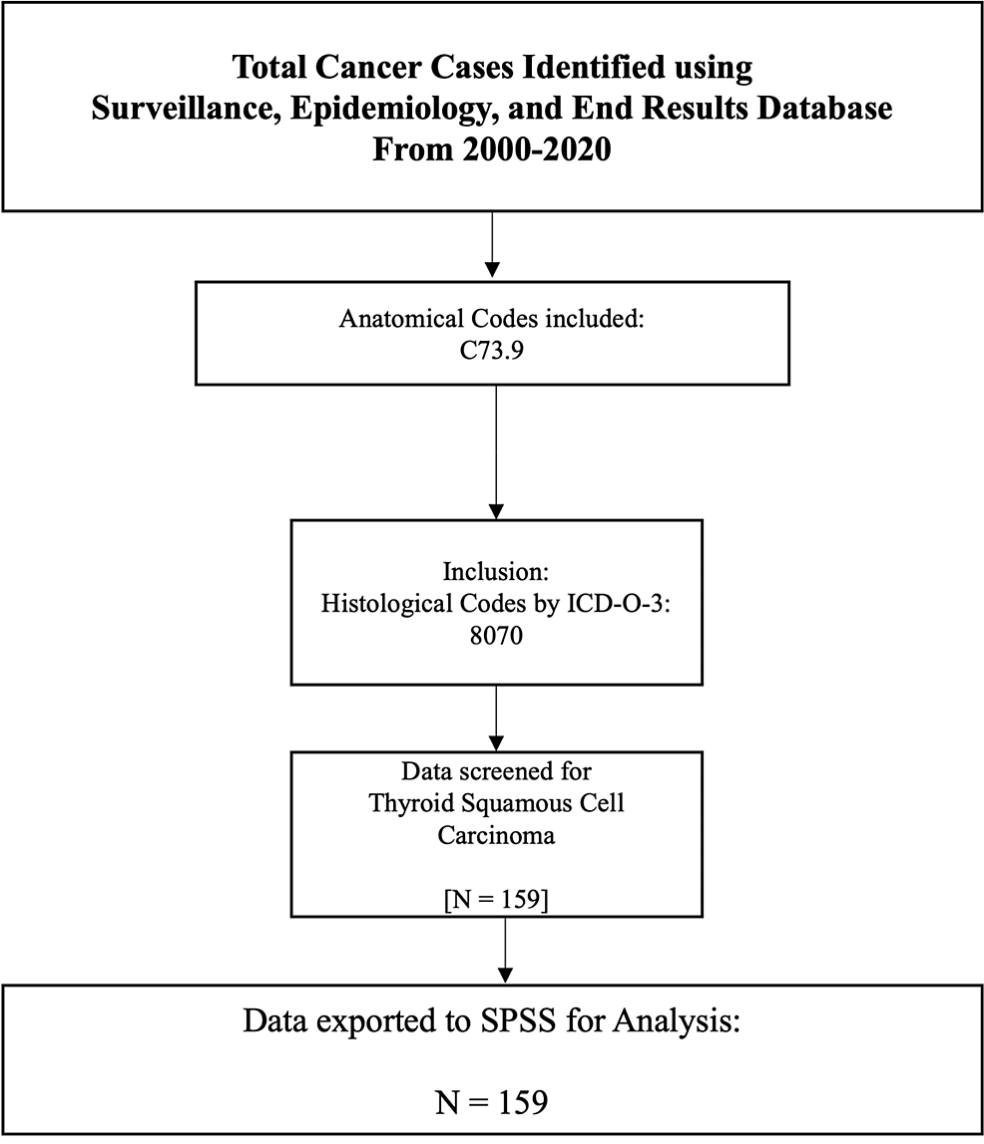
Inclusion and exclusion flowchart ICD-O-3, International Classification of Diseases Version 3

In 1973, the National Cancer Institute created SEER, a database providing data from institutions that cover around 26.5% of the United States population. Data from 2000 to 2020 were collected using the International Classification of Diseases Version 3 (ICD-O-3). The ICD topography code C73.9 and the ICD morphology code 8070 were used to query patients from SEER registries. The data were then exported to IBM SPSS Statistics for Windows, Version 28.0 (Released 2021; IBM Corp., Armonk, NY, USA) for analyses. Demographic data were extracted, including age, sex, race, income, and housing. Clinical characteristics included tumor size, tumor stage, nodal status, metastases, treatment modality, survival months, and the patient’s status. Any cases with unknown outcomes and missing death certificates were excluded. Median income was divided into less than $70,000 and greater than $70,000, based on the United States census, revealing that the 50th percentile was $70,181 in 2021 [8]. After screening for histology and anatomy, 159 cases were identified and exported to SPSS for analysis (Figure 1).

Statistical analysis

Cox proportional hazard regression analysis was used to search for associations between demographic factors, tumor characteristics, treatment modality, and OS. The SPSS software estimates log-rank p values, HRs, and 95% CIs. Such differences were visualized on a graph by generating Kaplan-Meier survival plots. The median follow-up in years was calculated using the reverse Kaplan-Meier method, with censure coded as the event of death. Univariate and multivariate survival analyses were used to view associations and search for independence in demographic, clinical, and treatment variables. In multivariate analysis, cases with unknown variables were censored. Any p-values <0.05 were considered statistically significant.

## Results

In this study, 159 cases of primary SCCT were identified.

Demographic data

Among the patients in this study, the median age was 71 ± 21 years. The IQR was 21. The median overall follow-up was 6.0 years. The most common age band was 80-89 years (n = 45, 28.3%), followed by 60-69 years (n = 40, 25.2%). This cohort had a female predominance, with 83 females (52.2%) and 76 males (47.8%). Regarding race, most cases were White (n = 108, 67.9%), followed by Hispanic (n = 19, 11.9%) and Asian or Pacific Islanders (n = 19, 11.9%). A total of 57.2% of the cohort was in the less than $70,000 annual income group, while 42.8% were in the greater than $70,000 group. Most of the cohort (89.3%) lived in urban areas, while the remainder (10.7%) lived in rural areas (Table 1).

**Table 1:** Demographic profiles, tumor characteristics, and treatment ^x^ The percentage (%) is calculated by dividing the number of cases (N) by the total number of known cases, providing real-time percentages. Note that unknown cases are not shown in the table. SCCT, squamous cell carcinoma of the thyroid

Characteristics, n = 159 (% of known cases^x^)	SCCT
Age, N (%)	
Median ± IQR	71 ± 21
0-69	77 (48.4%)
>69	82 (51.6%)
Sex, N (%)	
Female	83 (52.2%)
Male	76 (47.8%)
Ethnicity, N (%)	
White	108 (67.9%)
African American	12 (7.5%)
Asian or Pacific Islander	19 (11.9%)
Hispanic	19 (11.9%)
American Indian/Alaskan Native	0 (0.0%)
Tumor stage, N (%)	
Localized	12 (9.8%)
Regional	30 (24.4%)
Distant	81 (65.9%)
Tumor size, N (%)	
≤2.0 cm	10 (10.9%)
2.1-4 cm	18 (19.6%)
>4.0 cm	64 (69.6%)
Lymph node status, N (%)	
Positive	32 (66.7%)
Negative	16 (33.3%)
Metastasis, N (%)	
Yes	20 (22.0%)
Isolated	17 (85.0%)
No	71 (78.0%)
Treatment modality, N (%)	
Surgery (%)	52 (32.7%)
Radiation (%)	38 (23.9%)
Chemotherapy (%)	52 (32.7%)
Income, N (%)	
< $70,000	91 (57.2%)
> $70,000	68 (42.8%)
Housing, N (%)	
Urban	142 (89.3%)
Rural	17 (10.7%)

Tumor characteristics

When the extent of the disease was known (77.4%), most cases (n = 81, 65.9%) had distant metastasis, followed by regional spread (n = 30, 24.4%) and then localized disease (n = 12, 9.8%). The extent of the disease was unknown in 36 (22.6%) cases. Tumor size was known in 92 (57.9%) cases, of which 10 (10.9%) were ≤2 cm, 18 (19.6%) were 2.1-4.0 cm, and 64 (69.6%) were >4.0 cm. Lymph node status was known in 48 (30.2%) cases, of which 32 (66.7%) had positive lymph nodes. Distant metastasis was seen in 91 (57.2%) cases, of which 20 (22.0%) had a positive metastasis to the brain, bone, liver, or lung. Of these, most metastases were isolated (n = 17, 85.0%) (Table 1).

Treatment characteristics

**Figure 2:**
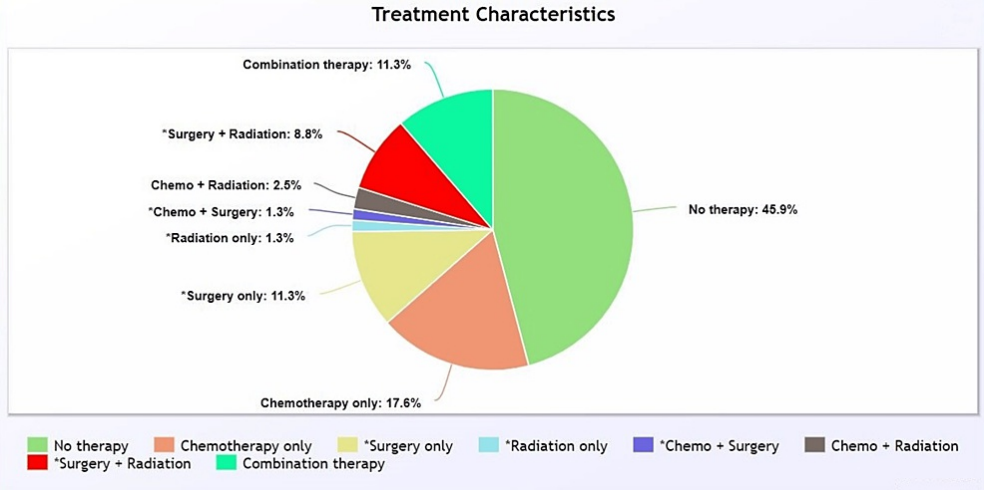
Pie chart of treatment modalities ^* ^Chemotherapy status is unknown

Most cases in this cohort underwent no therapy (n = 73, 45.9%). Of the total cases, 18 (11.3%) underwent combination therapy (surgery, radiation, and chemotherapy). Of the total cases, 28 (17.6%) received chemotherapy only, two (1.3%) received radiation only, and 18 (11.3%) underwent surgery only (Figure 2, Table 1).

Survival Analysis by Demographic Factors

**Figure 3:**
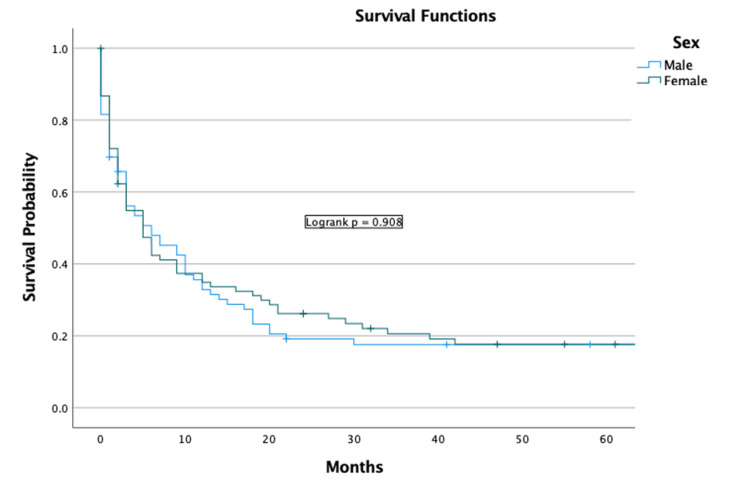
Sex Kaplan-Meier plot

**Figure 4:**
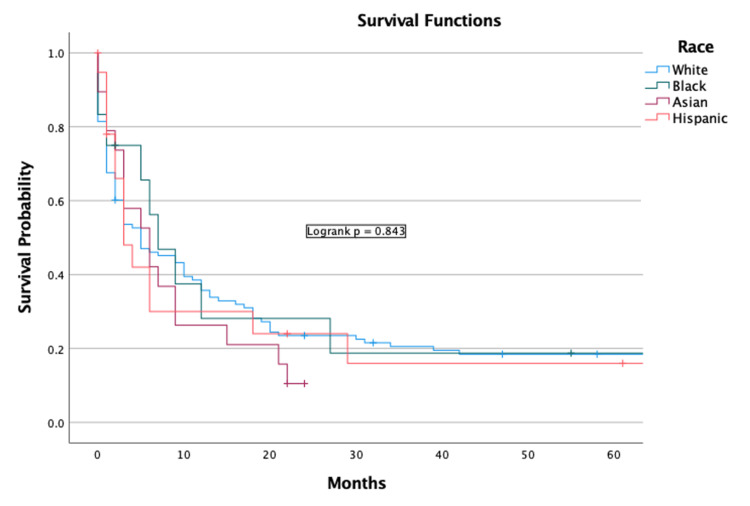
Race Kaplan-Meier plot

Univariate analysis indicated that increased age in terms of years was a negative predictor (OR = 1.04 (CI = 1.03-1.06), p < 0.001) (Table 2). Sex and race were not predictors of survival (Figures 3, 4).

**Table 2:** Univariate and multivariate analysis for factors contributing to OS ^* ^Significant for being associated with worse outcomes ^+^ Significant for being associated with better outcomes OS, overall survival

		Univariable		Multivariable	
Variables		HR (95% CI)	p-value	HR (95% CI)	p-value
Age	Increasing	Reference	1	1	1
		1.04 (1.03-1.06)	<0.001^*^	1.05 (1.03-1.06)	<0.001^*^
Stage	Local	Reference	1	1	1
	Regional	1.24 (0.56-2.76)	0.602	0.90 (0.40-2.02)	0.789
	Distant	2.66 (1.27-5.53)	0.009^*^	2.13 (1.02-4.48)	0.046^*^
Nodal status	Negative	Reference	1	1	1
	Positive	2.80 (1.24-6.35)	0.013^*^	1.55 (0.63-3.81)	0.338
Surgery	Did not undergo	Reference	1	1	1
	Underwent	0.44 (0.29-0.64)	<0.001^+^	0.59 (0.25-1.40)	0.192
Radiation	Did not undergo	Reference	1	1	1
	Underwent	0.54 (0.36-0.83)	0.004^+^	0.62 (0.26-1.48)	0.28
Chemotherapy	Did not undergo	Reference	1	1	1
	Underwent	0.86 (0.60-1.24)	0.421	0.51 (0.22-1.20)	0.124

Survival Analysis of Tumor Characteristics

The tumor stage was found to be a prognostic predictor of survival (p < 0.001). Univariate analysis revealed that distant dissemination of disease in terms of stage (OR = 2.66 (CI = 1.27-5.53), p = 0.009) was considered a negative predictor of prognosis (Table 2). Nodal status was also found to be a prognostic factor (p = 0.008), with univariate analysis revealing that a positive nodal status (OR = 2.80 (CI = 1.24-6.35), p = 0.013) was associated with worse outcomes.

Overall outcomes and survival analysis

**Figure 5:**
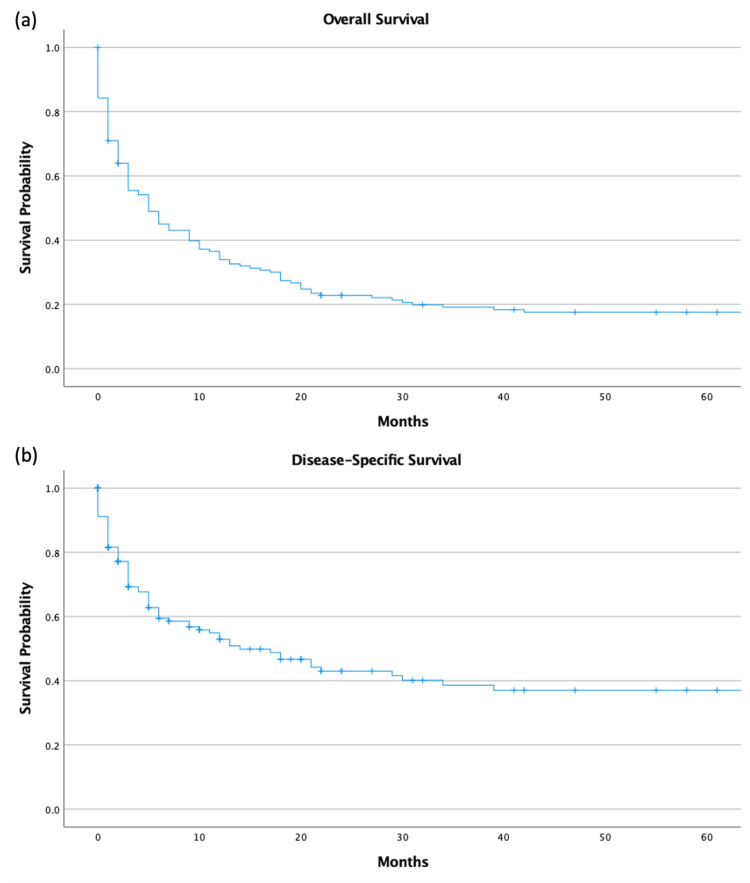
(a) OS and (b) DSS DSS, disease-specific survival; OS, overall survival

**Figure 6:**
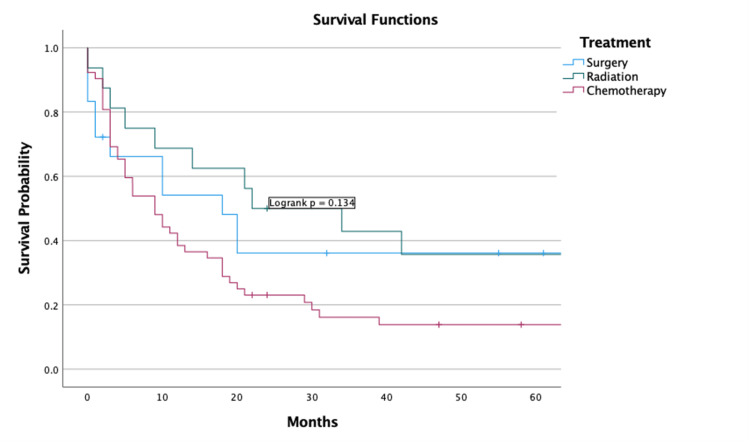
Survival analysis of treatment modality

The five-year OS of the study group was 17.6% (95% CI = 14.5-20.7). The five-year DSS was 37.6% (95% CI = 32.7-42.5). A Kaplan-Meier graph visualizing the five-year survival is listed below (Figure 5a, 5b). There was no significant difference regarding whether one underwent surgery, chemotherapy, or radiation (p = 0.134) (Figure 6). Univariate analysis revealed that undergoing radiation (HR = 0.54 (CI = 0.36-0.83), p = 0.004) and surgery (HR = 0.44 (CI = 0.29-0.64), p < 0.001) were positive predictors of survival in terms of whether they underwent the particular treatment or not. Chemotherapy did not affect survival (Table 2).

Multivariate analysis

Multivariable Cox proportional hazards analysis identified increasing age (OR = 1.05 (CI = 1.03-1.06), p < 0.001) and distant stage (OR = 2.13 (CI = 1.02-4.48), p = 0.046) as negative predictors for survival (Table 2).

## Discussion

SCCT is a rare type of thyroid carcinoma that accounts for less than 0.5% of primary thyroid cancer cases. Over the past few decades, as more extensive knowledge was attained, SCCT was listed in the “Other Carcinoma” in the second edition of the WHO Histological Typing of Thyroid Tumors in 1988 and later became a separate entity in the third and fourth editions [9-11]. However, the fifth version of the WHO Blue Books of Endocrine and Neuroendocrine Tumors categorizes thyroid squamous cell carcinoma as being under the umbrella of atypical thyroid carcinoma (ATC), which was further supported in the retrospective cohort study conducted by Xu et al. [2,3,12]. According to Xu et al., ATC and SCCT share similar squamous phenotypes, outcomes, and five-year OS rates, as well as usually being positive for PAX8 and BRAFV600E6.12. Furthermore, age, staging, leukocytosis, nodal status, and distant metastasis also negatively affect the prognosis of the two cancer types [13-17]. Clinically, SCCT and ATC both present with rapidly invading neck mass with dysphagia and hoarseness, cervical lymphadenopathy, and paraneoplastic manifestations [5,18,19]. ATC can also demonstrate squamous cell differentiation. As such, it is crucial to rule out ATC before diagnosing patients with primary SCCT.

While ATC and SCCT share various characteristics and outcomes, the results from previous publications demonstrated different findings on SCCT from descriptions of ATC in the WHO Blue Books. Based on our results and the results from Yang et al., sex, race, annual income, or housing were not significant prognostic factors for SCCT [6]. Additionally, our study did not show tumor size as a negative prognostic factor; notably, the average tumor size for SCCT (54 mm) is significantly smaller than the average size for ATC (80 mm) [5,6,20]. In contrast to these results, the WHO Blue Books state that male gender, large tumor sizes, non-white patients, and high-poverty areas negatively influence the prognosis for ATC [2]. In addition to differences in negative prognostic factors, the 1-year OS rate for squamous cell carcinoma (22.8%) is much lower than the one-year OS rate in ATC, which ranges from 38% to 63% [12].

Regarding differences in immunohistochemical features, paired-box gene 8 (PAX-8), thyroid transcription factor 1, and thyroglobulin are sensitive thyroid lineage markers. ATC and SCCT favor the PAX-8 marker [21]. However, the BRAF mutation has only appeared consistently in ATC. In SCCT, only two cases were reported relating to the BRAF mutation, and one of the cases indicated a positive BRAF mutation [22,23]. BRAF T1799A transversion mutation in exon 15 results in a V600E amino acid substitution activating BRAF kinase that functions in tumorigenesis [24]. It was the first and only case of BRAF mutation in primary SCCT over a decade ago [22]. More genomic work to explore molecular pathogenesis is needed for future studies.

Additionally, the findings from our analysis, indicating a five-year OS rate of 17.6% and a DSS rate of 37.6%, highlight the malignant potential of SCCT. These survival rates are alarmingly low, especially in comparison with the more common types of thyroid cancer, such as papillary and follicular carcinomas [22]. The lack of significant differences in survival among patients undergoing surgery, chemotherapy, or radiation highlights crucial gaps in treatment modalities for SCCT. This gap calls for reevaluating current treatment protocols and stresses the urgent need for novel therapeutic strategies.

Identifying age, tumor stage, nodal status, and distant metastases as negative prognostic factors aligns with oncological principles observed in other cancer types. These factors traditionally signify advanced disease or a higher tumor burden, both of which are known to be associated with poorer outcomes. Conversely, the finding that sex, race, income, and housing do not significantly influence survival is intriguing, suggesting that the biological aggressiveness of SCCT may overshadow the socioeconomic and demographic variables often associated with cancer outcomes. This insight may redirect research focus toward understanding the molecular and genetic basis of SCCT, potentially paving the way for targeted therapies.

The limitations of this study are common to similar large retrospective observational database studies in terms of missing or incomplete information, misclassification of variables, and errors in the information entered in the database. Low-quality or incomplete data is most likely to affect low-incidence variables. The study results may also be adversely impacted by a lack of consistent definitions, particularly regarding tumor grade and changes over time in tumor classification. SEER does not provide information about the specific type of surgery or indications regarding the surgery.

## Conclusions

SCCT typically presents in older adults and has a slight female predilection. The data underscore the aggressive nature of SCCT, with most patients presenting with advanced disease and demonstrating poor responses to conventional treatments like chemotherapy. However, radiation and surgery have shown potential benefits, highlighting the importance of early detection and personalized treatment. Age and advanced disease stage were identified as negative survival predictors, emphasizing the critical need for early diagnosis and the exploration of novel therapeutic strategies to improve outcomes in SCCT.

## References

[1] Li W (2022). The 5th edition of the World Health Organization classification of hematolymphoid tumors. Leukemia [Internet].

[2] Basolo F, Macerola E, Poma AM, Torregrossa L (2023). The 5th edition of WHO classification of tumors of endocrine organs: changes in the diagnosis of follicular-derived thyroid carcinoma. Endocrine.

[3] Baloch ZW, Asa SL, Barletta JA (2022). Overview of the 2022 WHO Classification of Thyroid Neoplasms. Endocr Pathol.

[4] Lloyd RV, Osamura RY, Klöppel G, Rosai J (2017). WHO Classification of Tumours of Endocrine Organs. Rosai J.

[5] Lam KY, Lo CY, Chan KW, Wan KY (2000). Insular and anaplastic carcinoma of the thyroid: a 45-year comparative study at a single institution and a review of the significance of p53 and p21. Ann Surg.

[6] Yang S, Li C, Shi X (2019). Primary squamous cell carcinoma in the thyroid gland: a population-based analysis using the SEER database. World J Surg.

[7] Au JK, Alonso J, Kuan EC, Arshi A, St John MA (2017). Primary squamous cell carcinoma of the thyroid: a population-based analysis. Otolaryngol Head Neck Surg.

[8] Kollar JS and M (2022). Income in the United States: 2021. Income in the United States.

[9] Hedinger C, Williams ED, Sobin LH (1993). Other carcinomas. World Health Organization International Histological Classification of Tumours: Histological Typing of Thyroid Tumours, Second Edition.

[10] Lam KY, Sakamoto A (2004). Squamous cell carcinoma. World Health Organization Classification of Tumours: Pathology and Genetics - Tumours of Endocrine Organs.

[11] Carcangiu ML, Lam AK, Montone KT (2017). Squamous cell carcinoma. WHO Classification of Tumours of Endocrine Organs, Fourth Edition.

[12] Xu B, Fuchs T, Dogan S (2020). Dissecting anaplastic thyroid carcinoma: a comprehensive clinical, histologic, immunophenotypic, and molecular study of 360 cases. Thyroid.

[13] Sugitani I, Miyauchi A, Sugino K, Okamoto T, Yoshida A, Suzuki S (2012). Prognostic factors and treatment outcomes for anaplastic thyroid carcinoma: ATC Research Consortium of Japan cohort study of 677 patients. World J Surg.

[14] Lin B, Ma H, Ma M (2019). The incidence and survival analysis for anaplastic thyroid cancer: a SEER database analysis. Am J Transl Res.

[15] Janz TA, Neskey DM, Nguyen SA, Lentsch EJ (2019). Is the incidence of anaplastic thyroid cancer increasing: a population based epidemiology study. World J Otorhinolaryngol Head Neck Surg.

[16] Hvilsom GB, Londero SC, Hahn CH (2018). Anaplastic thyroid carcinoma in Denmark 1996-2012: a national prospective study of 219 patients. Cancer Epidemiol.

[17] Glaser SM, Mandish SF, Gill BS, Balasubramani GK, Clump DA, Beriwal S (2016). Anaplastic thyroid cancer: prognostic factors, patterns of care, and overall survival. Head Neck.

[18] Saito K, Kuratomi Y, Yamamoto K (1981). Primary squamous cell carcinoma of the thyroid associated with marked leukocytosis and hypercalcemia. Cancer.

[19] Yazawa S, Toshimori H, Nakatsuru K, Katakami H, Takemura J, Matsukura S (1995). Thyroid anaplastic carcinoma producing granulocyte-colony-stimulating factor and parathyroid hormone-related protein. Intern Med.

[20] Limberg J, Ullmann TM, Stefanova D, Finnerty BM, Beninato T, Fahey TJ 3rd, Zarnegar R (2020). Prognostic characteristics of primary squamous cell carcinoma of the thyroid: a national cancer database analysis. World J Surg.

[21] Lai WA, Hang JF, Liu CY (2020). PAX8 expression in anaplastic thyroid carcinoma is less than those reported in early studies: a multi-institutional study of 182 cases using the monoclonal antibody MRQ-50. Virchows Arch.

[22] Ko YS, Hwang TS, Han HS, Lim SD, Kim WS, Oh SY (2012). Primary pure squamous cell carcinoma of the thyroid: report and histogenic consideration of a case involving a BRAF mutation. Pathol Int.

[23] Chu TP, Chen WC, Wang TY, Cheng SP (2016). Genetic alterations in primary squamous cell carcinoma of the thyroid. Pathology.

[24] Xing M (2005). BRAF mutation in thyroid cancer. Endocr Relat Cancer.

